# Folate-Modified Albumin-Functionalized Iron Oxide Nanoparticles for Theranostics: Engineering and In Vitro PDT Treatment of Breast Cancer Cell Lines

**DOI:** 10.3390/pharmaceutics17080982

**Published:** 2025-07-30

**Authors:** Anna V. Bychkova, Maria G. Gorobets, Anna V. Toroptseva, Alina A. Markova, Minh Tuan Nguyen, Yulia L. Volodina, Margarita A. Gradova, Madina I. Abdullina, Oksana A. Mayorova, Valery V. Kasparov, Vadim S. Pokrovsky, Anton V. Kolotaev, Derenik S. Khachatryan

**Affiliations:** 1 Emanuel Institute of Biochemical Physics of Russian Academy of Sciences, 4, Kosygina Str., Moscow 119334, Russia; maria.g.gorobets@gmail.com (M.G.G.); stropdiva@yandex.ru (A.V.T.); alenmark25@gmail.com (A.A.M.); tuantonyx@yahoo.com (M.T.N.); triyozhika@gmail.com (M.I.A.); vvkas@yandex.ru (V.V.K.); vadimpokrovsky@gmail.com (V.S.P.); kolotaev2005@rambler.ru (A.V.K.); derenik-s@yandex.ru (D.S.K.); 2 N.N. Blokhin National Medical Research Center of Oncology, 24, Kashirskoye Sh., Moscow 115478, Russia; 3 N.N. Semenov Federal Research Center for Chemical Physics, Russian Academy of Sciences, 4, Kosygina Str., Moscow 119991, Russia; m.a.gradova@gmail.com; 4 Science Medical Center, Saratov State University, 83 Astrakhanskaya Str., Saratov 410012, Russia; oksanaamayorova@gmail.com; 5 Department of Biochemistry, Institute of Medicine, RUDN University, 6, Miklukho-Maklaya Str., Moscow 117198, Russia

**Keywords:** human serum albumin (HSA), iron oxide nanoparticles (IONPs), photodynamic therapy (PDT), hybrid magnetic nanosystems, folic acid, methylene blue, theranostics

## Abstract

**Background/Objectives**: Magnetic iron oxide nanoparticles (IONPs), human serum albumin (HSA) and folic acid (FA) are prospective components for hybrid nanosystems for various biomedical applications. The magnetic nanosystems FA-HSA@IONPs (FAMs) containing IONPs, HSA, and FA residue are engineered in the study. **Methods**: Composition, stability and integrity of the coating, and peroxidase-like activity of FAMs are characterized using UV/Vis spectrophotometry (colorimetric test using o-phenylenediamine (OPD), Bradford protein assay, etc.), spectrofluorimetry, dynamic light scattering (DLS) and electron magnetic resonance (EMR). The selectivity of the FAMs accumulation in cancer cells is analyzed using flow cytometry and confocal laser scanning microscopy. **Results**: FAMs (d_N_~55 nm by DLS) as a drug delivery platform have been administered to cancer cells (human breast adenocarcinoma MCF-7 and MDA-MB-231 cell lines) in vitro. Methylene blue, as a model photosensitizer, has been non-covalently bound to FAMs. An increase in photoinduced cytotoxicity has been found upon excitation of the photosensitizer bound to the coating of FAMs compared to the single photosensitizer at equivalent concentrations. The suitability of the nanosystems for photodynamic therapy has been confirmed. **Conclusions**: FAMs are able to effectively enter cells with increased folate receptor expression and thus allow antitumor photosensitizers to be delivered to cells without any loss of their in vitro photodynamic efficiency. Therapeutic and diagnostic applications of FAMs in oncology are discussed.

## 1. Introduction

Photodynamic therapy (PDT) is an effective method of cell destruction, including both cancer and bacterial cells. In recent years, the systems for delivery, particularly targeted delivery, of photosensitizers (PSs) for PDT [1,2,3,4,5,6,7] and the use of PDT for the treatment of epithelial tumors and visceral cancers have been actively explored. The PDT method is based on the generation of singlet oxygen or other reactive oxygen species (ROS) by photoexcited PS molecules in the presence of molecular oxygen. The PDT efficiency is determined both by physicochemical characteristics of the PS itself (i.e., intense absorption in the red part of the visible spectrum, which shows better penetration into tissues, efficient conversion to the triplet state, high singlet oxygen quantum yields, low dark phototoxicity, resistance to aggregation in biological media, etc.), and the selectivity of PS accumulation in the tumor area. The targeted delivery of PSs reduces adverse effects on healthy cells, the period of photosensitization during PDT, and the effective dose of the drug to facilitate excretion [8].

Currently, various innovative delivery systems are being developed for advanced applications in tumor therapy and imaging. In particular, researchers have focused on the development of hybrid systems based on magnetic nanoparticles (MNPs) for the treatment and diagnosis (theranostics) of cancer. The most widely used MNPs for biomedical applications are iron oxide nanoparticles (IONPs) [9,10]. Human serum albumin (HSA), an abundant plasma protein, which is an important carrier protein in the blood for endogenous and exogenous molecules, is often used as a component of the artificial protein coatings on the surface of different nanoparticles, demonstrating stability, high biocompatibility, moderate toxic effects, low-to-negligible immunogenicity, and biodegradability; reducing toxicity of certain drugs and lowering their clearance rates; increasing circulatory half-life of drugs and their solubility in plasma [11,12,13]. Albumin also enhances ingestion of drugs into lysosomal compartments of cancer cells compared to normal cells [14] and has a natural ability to accumulate in areas of inflammation [11]. The hybrid nanosystems composed of MNPs (particularly IONPs) and HSA can be used for different medical applications: targeted delivery of biologically active substances and drugs (passive targeting due to the effect of increased vascular permeability of the tumor vessels, active targeting using biovectors or magnetic field retention capabilities) and advanced release of drugs at the target location; photothermal therapy (PTT) and magnetic hyperthermia to tumors; the contrast enhancement in magnetic resonance imaging (MRI) of tumor tissues [15,16,17,18,19,20,21,22]. Recently, multiple areas of medical application of IONPs with enzyme-like, particularly, peroxidase-like activity (nanozymes, IONzymes) have been proposed [23,24]. Also, albumin-coated IONPs with controlled peroxidase-like activity have been engineered and tested in vivo as a contrasting agent for computed tomography [17]. In 2016, it was shown that the surface of IONPs, characterized by the presence of Fe^2+^, can cause cancer cell death by the mechanism of ferroptosis [25], which was discovered before and included lipid peroxidation of cell membranes due to the Fenton reaction [26]. IONzymes can be used in hypoxia zones [27] and reduce the resistance of tumors to radio- and chemotherapy [28]. Nowadays, IONPs are being discussed as a basis for the design and fabrication of innovative H_2_O_2_-driven nanoreactors with catalytic performances to convert H_2_O_2_ into more cytotoxic ROS [27]. Nanoreactors for chemodynamic therapy (CDT) have been found to be efficient in selective inhibition of tumor growth both independently and in rational combination with other treatments [27,29].

In recent years, there have been many studies aimed at using magnetic nanoparticles as delivery vehicles of PSs for PDT [30,31,32], which in some cases has been combined with the delivery of other biologically active substances. Various classes of PSs, particularly porphyrins [33], phthalocyanines [34], chlorins [35], bacteriochlorins [36], curcumin [37], and cyanine dyes [38] have been used as part of magnetic nanosystems for theranostics and combined PDT and PTT. Most studies implement covalent binding of the drug to the surface of nanoparticles [35,39,40], which increases stability of the composition, requires additional steps in the synthesis of the composition, and development of strategies for controlled release of the drug. However, recently, non-covalent approaches to the functionalization of MNPs with PSs have become predominant [36,37,38,41]. The obtained nanosystems demonstrate high diagnostic accuracy and therapeutic activity both in vitro and in vivo. Moreover, the non-covalent approach requires significantly less time and reagents compared with the covalent crosslinking of fluorophores with the nanoparticle surface. The coating on nanoparticles provides effective binding of the PS/fluorophore molecules, preventing their aggregation in aqueous solution. Generally, some of the hydrophobic PS molecules bind effectively to albumin [42,43,44,45], which makes it possible to use the surface of albumin-modified MNPs for PS immobilization. Thus, in different studies [36,46] modification of magnetic nanoparticles with PSs was carried out directly due to the non-covalent binding of PS molecules with the particle surface, notably with HSA.

At present, the high potential of the active drug delivery strategies using nanoparticles modified with biologically active substances is considered undeniable. Active drug transport creates opportunities to reduce the toxic effects of drugs on the whole body and provides prolonged action of drugs [47]. Modern biovector targeting strategies involve the use of antibodies, aptamers, peptides, and low molecular weight compounds [48] to bind nanosystems to biological targets on the walls of blood vessels and on cancer cells. Folates are necessary for the active proliferation of cancer cells [49]. Thus, folic acid (folate) receptors are highly selective targets on various cancer cells compared with normal cells. Conjugates that have the folic acid (FA) residue in their structure serve to transport drugs and contribute to the visualization of tumors without exerting immunotoxic effects [50]. Drugs that include FA residues have reached clinical trials. The efficiency of FA as a targeting agent (biovector) has been demonstrated on many cell lines [51,52]. Folate-conjugated nanosystems for tumor theranostics include those based on MNPs [53], IONPs, and serum albumin [54]. Folate-modified nanoparticles with albumin-functionalized surfaces have exhibited the potential for use in PDT [55,56].

In our previous studies [17,57] we have developed an original protocol of obtaining a magnetically responsive drug delivery platform based on IONPs with a stable albumin coating. The goals of the present study were as follows: (1) to engineer a stable HSA coating on iron oxide nanoparticles and to modify it with the folic acid residue; (2) to bind a model PS to the surface of the obtained systems; (3) to characterize the coating (its composition and thickness, stability and integrity) of hybrid nanosystems and their peroxidase-like activity using physical-chemical methods (UV/Vis spectrophotometry, spectrofluorimetry, dynamic light scattering (DLS) and electron magnetic resonance (EMR) spectroscopy); (4) to confirm targeting of hybrid nanosystems into cancer cells (MCF-7 and MDA-MB-231) in vitro; and (5) to show the effectiveness of the hybrid particles as a drug delivery platform for the photosensitizer non-covalently bound to their surface. Although numerous studies have described the hybrid nanosystems containing two or three components, including IONPs, HSA, FA, or PS, such a complex quaternary system, employing the advantages of all these components, to the best of our knowledge, has been engineered for the first time.

## 2. Materials and Methods

### 2.1. Preparation of Albumin-Functionalized Magnetic Iron Oxide Nanoparticles

Magnetic IONPs were fabricated from FeSO_4_ × 7H_2_O and FeCl_3_ × 6H_2_O using the co-precipitation method and electrostatically stabilized with 0.1 M phosphate-citrate buffer (0.05 M NaCl) with pH 4.3 as described in previous studies [17,58]. The structural, magnetic, and electronic characteristics of the particles have been studied before [58]. IONP hydrosol was preserved in a sealed vessel. The catalytic/IONzyme properties of IONPs prior to sample preparation were confirmed as described previously [17].

**Figure 1:**
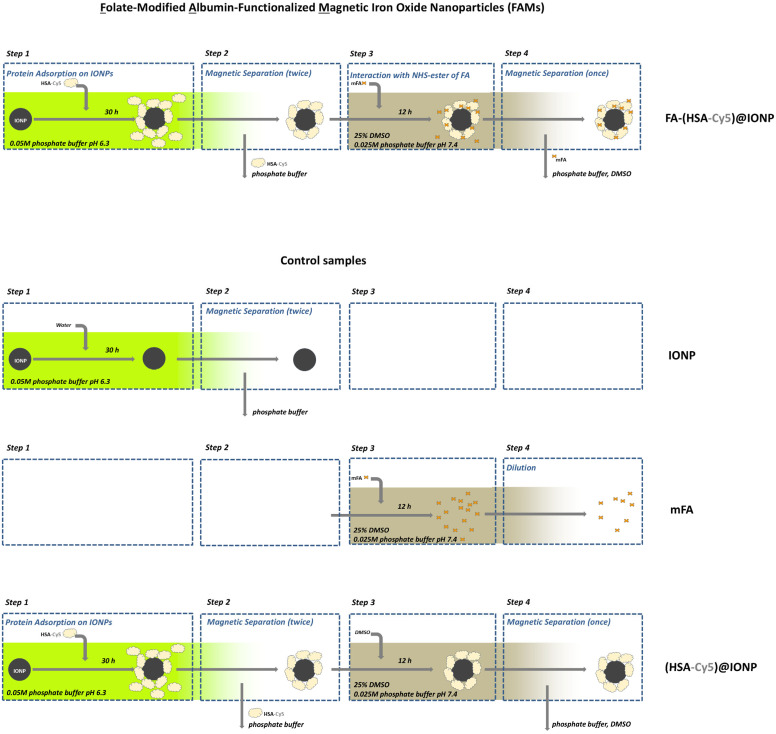
The scheme of preparation of the folate-modified albumin-functionalized magnetic iron oxide nanoparticles (FAMs) and the control samples.

The 0.05 M phosphate buffer pH 6.3 for HSA coating formation was chosen according to our previous study [59]. IONPs hydrosol was dissolved 20-fold in the buffer and incubated on Nd-Fe-B magnets for removal of the largest particles from the solution before HSA addition. An aqueous solution of HSA (A1653, Sigma) was added to the IONPs hydrosol immediately as 10 vol. % under stirring on Vortex V-1 plus (Biosan, Riga, Latvia). The concentration of IONPs and HSA in the mixture was 0.38 mg/mL and 3 mg/mL, respectively (the C_HSA_/C_IONPs_ ratio was ~8 [mg/mg]). The samples of IONPs and HSA were incubated for 30 h at 25 °C in Rotator Multi Bio RS-24 (Biosan, Riga, Latvia). After incubation, magnetic separation was used to wash out the excess components (for example, the buffer and the protein at Step 2, see Figure 1). Sterile water for injections with 5 mM NaCl was added after the removal of the excess components due to the magnetic separation. For some experiments HSA was conjugated with fluorescent dye (details are in Section 2.3) and used for the preparation of functionalized magnetic iron oxide nanoparticles for further analysis.

The complete removal of the protein excess (in supernatants) and the protein amount on the surface of IONPs was examined through measuring the amount of protein using UV/Vis spectroscopy and Bradford protein assay (details are in Section 2.12). Particle size data were collected for the samples before and after magnetic separation using dynamic light scattering (details are in Section 2.10). Electron magnetic resonance techniques (details are in Section 2.11) were used to evaluate concentrations of IONPs in different samples. Concentrations of the functionalized IONPs are given as the corresponding IONP concentrations and/or protein concentrations in the text below.

### 2.2. Protein Coating Stability and Integrity Analysis

The addition of human immunoglobulin G (IgG) and HSA was used to check the stability and integrity of the HSA coating on IONPs obtained as described in Section 2.1.

IgG was purchased from the Scientific and Production Association for Immunological Preparations “Microgen” (Russia) and initially treated as described earlier [17,59,60]. As it has been shown, adsorption of IgG molecules on the IONPs surface depends on the presence and stability of the HSA coating. This effect can be explained by the relatively high affinity of IgG for the nanoparticle surface [61].

75 µL of IgG (0.66 mg/mL) and HSA (3.3 and 33 mg/mL) were added to 150 µL of the functionalized IONPs with IONPs concentration ~0.7 mg/mL and incubated overnight in 0.05 M phosphate buffer pH 6.3. The supernatants after magnetic separation of the samples incubated with IgG and HSA were then analyzed via absorption (details are in Section 2.12) and fluorescence (details are in Section 2.13) spectroscopy. The integrity of the HSA coating was evaluated by desorption of HSA from IONPs obtained from the intensity of Cy5 spectra of the analyzed supernatants.

Electron magnetic resonance techniques (details are in Section 2.11) were used to reveal IgG interaction with IONPs with and without the HSA coating. The samples were analyzed to detect the stability and integrity of HSA coatings on IONPs. The final C_IgG_/C_IONPs_ ratio in solutions reached 2.6 [mg/mg] and was equivalent in the samples taken for comparison.

### 2.3. Preparation of Cy5-Conjugated HSA

HSA conjugated with fluorescent dye was used for the preparation of functionalized magnetic iron oxide nanoparticles (as described in Section 2.1). HSA was conjugated with the fluorescent dye Cyanine 5 NHS-ester (Lumiprobe, Moscow, Russia) by covalent crosslinking according to the protocol described before [62]. Namely, 400 µL of Cy5 solution in dimethyl sulfoxide (DMSO) (1 mg/mL) was gradually added dropwise to 1 mL of HSA solution (200 mg/mL) under vigorous stirring (1000 rpm). The mixture was incubated at 4 °C for 24 h under constant stirring. The unreacted Cy5 was removed by dialysis (M-Cel, Viskase, Beauvais, France, pore diameter 14 kDa) for 3 days at 4 °C against saline (0.15 M NaCl). The amount of Cy5 was analyzed from the absorbance maximum at 646 nm using a calibration in the Cy5 concentration range of 0.05–1.0 μg/mL with the CLARIO Star Plus Microplate reader (BMG Labtech, Ortenberg, Germany). The resulting solution of HSA-Cy5 contained 2.4 ± 0.6 μg/mL Cy5.

The albumin-functionalized IONPs obtained using HSA conjugated with Cy5 were used as described above (see Section 2.2) to analyze the integrity of the HSA coating under the action of IgG and additional HSA. Flow cytometry and confocal laser scanning microscopy were used to detect nanosystems into cells.

### 2.4. Preparation of Folate-Modified Albumin-Functionalized Magnetic Iron Oxide Nanoparticles (FAMs)

The N-hydroxy succinimide (NHS) ester of folic acid was prepared as it was described in [63,64,65,66]. Folic acid (FA) (3.53 g), NHS (1.77 g) and triethylamine (1.8 mL) were dissolved in dimethyl sulfoxide (70 mL) at 30 °C. The reaction mixture was stirred at 30 °C until a clear solution was obtained. Then N-dicyclohexylcarbodiimide (3.32 g) was added, and the reaction mixture was stirred at room temperature for 48 h. The precipitate of dicyclohexylurea (DCU) was filtered off, and the yellow DMSO solution of FA-NHS was concentrated at 40–50 °C/1 mm Hg, and the obtained oil was triturated with diethyl ether (50 mL), and the resulting precipitate was filtered off. The yellow residue was again washed with diethyl ether (50 mL), filtered, and dried in a vacuum oven at RT to yield 4.3 g NHS-ester of folic acid (mFA) as a yellow powder with good solubility in DMSO. Physical-chemical characteristics for mFA correspond to those presented in the study [66].

**Scheme 1:**
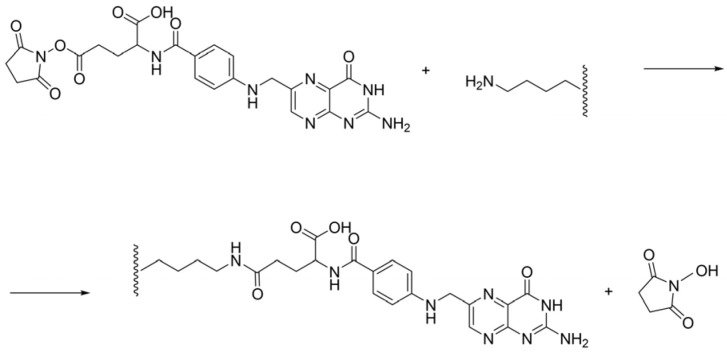
The binding reaction of NHS-ester of folic acid and HSA.

The binding reaction of mFA and HSA is a nucleophilic substitution reaction that proceeds through the available lysine residues of the albumin molecule (Scheme 1).

A solution of mFA in DMSO (3 mg/mL) was prepared 3–24 h before the experiments and incubated in the dark due to the FA photosensitivity [67,68,69]. Different pH and phosphate buffer concentrations from 0 to 25 mM (pH~7.4; pH~6.3) were tested to detect the binding between mFA and HSA and confirmed the possibility of using these conditions for carrying out a multi-step process (Figure 1) for the modification of albumin-functionalized IONPs in the presence of phosphate buffer from traces to 25 mM. According to other studies, the duration of mFA and serum albumin co-incubation is different, i.e., 3 min [70] or 24 h [71], so we varied the duration of mFA and HSA co-incubation in our experiments from 10 min to 48 h. The mFA/HSA ratio we varied from 1/2 to 60/1 [mol/mol] by changing the concentration of HSA or mFA. Since we considered it important to keep a relatively low percentage of DMSO in the reaction mixture containing HSA, it was equal to 25% in our experiments, which is close to the DMSO concentration in the study [72]. The control samples of mFA without HSA and HSA without mFA were prepared in a mixed solution equivalent to the solution in the reaction mixture. UV/Vis absorption spectra (see Section 2.12 for details) and fluorescence spectra (see Section 2.13 for details) of all samples were obtained after dilution in the mixed solution.

Experiments with different pH and buffer concentration, amounts of mFA and HSA, mFA and HSA co-incubation time, etc., resulted in the selection of suitable reaction conditions for obtaining FAMs. Namely, the reaction was carried out in 25 mM phosphate buffer pH~7.4 with 25% DMSO. The albumin-functionalized IONPs, after magnetic separation (performed twice), obtained as described in Section 2.1 (see Step 2 in Figure 1), were mixed with mFA. mFA/HSA ratio was taken from 14/1 to 28/1 [mol/mol] after evaluation of HSA amount in the sample. A control sample with the addition of DMSO instead of the mFA solution (the sample with zero mFA content) was also obtained (see Figure 1). Experimental and control samples were incubated overnight. After that, the additional magnetic separation was carried out, and the completeness of the protein excess removal was confirmed by the protein amount measurement in the supernatant obtained.

At further stages of the study, we used FAMs (FA-(HSA-Cy5)@IONPs or FA-HSA@IONPs), the control sample with zero FA content ((HSA-Cy5)@IONPs or HSA@IONPs), and additional control samples, namely FA, mFA, HSA, FA-HSA, IONPs. All the samples were stored for no longer than three days after their preparation before administration to cells.

### 2.5. Cancer Cell Cultures

Human breast adenocarcinoma MCF-7 and MDA-MB-231 cell lines were obtained from the American Type Culture Collection (Manassas, VA, USA). Cells were routinely propagated in Dulbecco’s modified Eagle medium (DMEM) supplemented with 2 mM *L*-glutamine, 100 U/mL penicillin, 100 µg/mL streptomycin (all from PanEco, Moscow, Russia), and 10% fetal bovine serum (HyClone, Logan, UT, USA) at 37 °C, 5% CO_2_ in a humidified atmosphere. Cells in the logarithmic phase of growth were used in the experiments.

### 2.6. Cellular Uptake of (HSA-Cy5)@IONPs and FA-(HSA-Cy5)@IONPs (FAMs)

MDA-MB-231 and MCF-7 cells grown in 300 µL DMEM in 24-well plates (Nunc, Denmark) were incubated with 30 µL (HSA-Cy5)@IONPs or FA-(HSA-Cy5)@IONPs (FAMs) for 1, 3, 6, and 24 h at 37 °C. After the completion of incubation, the cells were detached with Versene (PanEco, Russia) solution at 37 °C for 10 min and washed with phosphate buffer saline (PBS). Fluorescence of accumulated (HSA-Cy5)@IONPs or FA-(HSA-Cy5)@IONPs was analyzed with flow cytometry on a BD FACSCanto II (BD Biosciences, San Jose, CA, USA) in the APC (Allophycocyanin) channel. For each sample, 10,000 events were collected.

### 2.7. Intracellular Distribution of (HSA-Cy5)@IONPs and FA-(HSA-Cy5)@IONPs

MCF-7 and MDA-MB-231 cells were grown in 35 mm Petri dishes with a glass bottom (SPL, Pyeongtaek, Republic of Korea) until 50% confluence. Then, (HSA-Cy5)@IONPs and FA-(HSA-Cy5)@IONPs (final concentration 33.5 ± 4.5 µg/mL) were added for 3 h. Non-fluorescent components of nanosystems were excluded from analysis by monitoring cellular autofluorescence in response to the addition of IONPs, HSA@IONPs, FA-HSA@IONPs in equivalent final concentrations under similar experimental conditions. Microscopy settings were adjusted to only show Cy5 fluorescence.

After the completion of incubation, the cells were washed with saline and stained with the MitoTracker™ Green (0.5 µM, 20 min) (Invitrogen Corp., Carlsbad, CA, USA) according to the manufacturer’s recommendations. After staining, the cells were washed with saline and subjected to confocal laser scanning microscopy at the following wavelengths: Cy5 channel (λ_ex_ = 633 nm/λ_em_ = 650–750 nm), MitoTracker™ Green (λ_ex_ = 476 nm/λ_em_ = 490–560 nm) [73]. The confocal laser scanning microscope Leica TCS SP 5, equipped with Leica LAS X 5.0.2 software (Leica Microsystems GmbH, Wetzlar, Germany) was used for imaging.

### 2.8. Preparation of MB-HSA@IONPs and MB/FA-HSA@IONPs

Methylene blue (MB, Sigma-Aldrich, St. Louis, MI, USA) was dissolved in DMSO to prepare a 5 mM stock solution. Afterward, 1 μL MB (5 mM) was added to the 500 µL colloidal solution of HSA@IONPs or FA-HSA@IONPs. The mixture was incubated at room temperature for 12 h. Then, solutions of MB-HSA@IONPs and MB/FA-HSA@IONPs were obtained by magnetic separation (applied to remove the free MB) and redispersion in sterile water for injections.

The amount of MB loaded onto the particles (MB-HSA@IONPs and MB/FA-HSA@IONPs) was studied using a UV3101RS spectrophotometer (Shimadzu, Kyoto, Japan) to record the absorption of MB at 660 nm.

**Figure 2:**
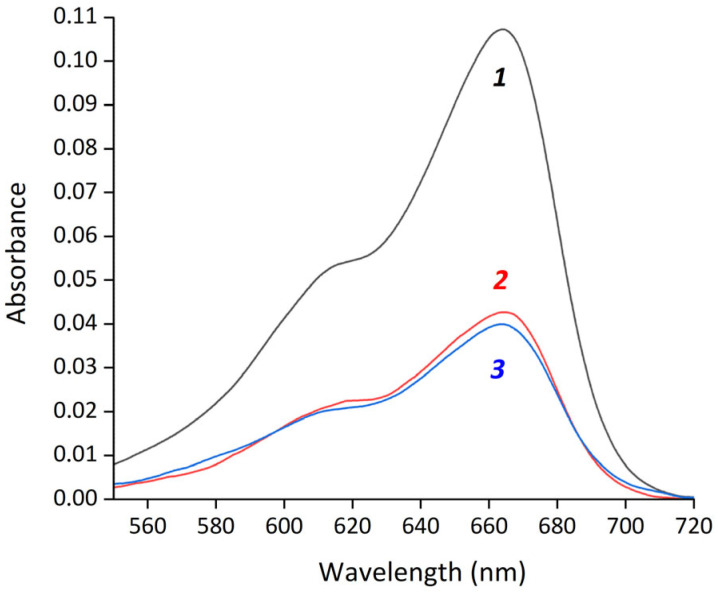
Absorption spectra of methylene blue in water (1) and supernatants after magnetic separation of MB-HSA@IONPs (2) and MB/FA-HSA@IONPs (3).

Encapsulation efficiency (EE%) of MB was calculated as follows:

EE% = (A_input_ − A_supernatant_) ÷ A_input_ × 100%,

where A_input_ is the absorbance of initial MB, and A_supernatant_ is the absorbance of MB in the supernatant after magnetic separation. EE% both for MB-HSA@IONPs and MB/FA-HSA@IONPs were 61.5 ± 1.5% (Figure 2). The measured MB binding constants with albumin were 4.38 × 10^4^ for HSA and 4.04 × 10^4^ for FA-HSA (Figure S1). The difference between these constants is less than 10%, which is within the experimental error and explains the similar EE% between MB-HSA@IONPs and MB/FA-HSA@IONPs.

Loading capacity (LC%) of MB-HSA@IONPs and MB/FA-HSA@IONPs was calculated as follows in Section 3.4:

m_MBloaded_ = m_input_ − m_supernatant_ = (A_input_ − A_supernatant_) ÷ A_input_ × m_input_ LC% = EE% × m_input_ ÷ m_IONPs_,

where m_Mbloaded_ and m_input_ are the masses of MB loaded onto IONPs and initially added to IONPs, respectively, and m_IONPs_ is the mass of the particles.

### 2.9. Cell Viability

MDA-MB-231 and MCF-7 cells grown in 200 µL DMEM in 96-well plates (Nunc, Denmark) were treated with 20 µL HSA@IONPs, FA-HSA@IONPs, MB-HSA@IONPs (final concentration 0.6 µM MB), and MB/FA-HSA@IONPs (final concentration 0.6 µM MB) for 3 h at 37 °C. MB at a final concentration of 0.6 µM was used as a reference photosensitizer. At the end of incubation, the cell medium was aspirated and replaced with 200 µL fresh medium. For phototoxicity studies, the cells were irradiated at a fluence rate of 21 mW/cm^2^ to a total fluence of 120 J/cm^2^ using a 660 nm laser (AFC Polironics, Moscow, Russia) and incubated for an additional 24 h at 37 °C. For dark cytotoxicity studies, the cells were incubated for 24 h at 37 °C.

After that, 20 µL of 5 mg/mL tetrazolium dye MTT (3-(4,5-dimethylthiazol-2-yl)-2,5-diphenyltetrazolium bromide) (PanEco, Moscow, Russia) was added to each well and incubated for 1 h at 37 °C. Subsequently, the medium was removed and 100 µL DMSO was added to solubilize formazan. The optical density of formazan solution was read on a Multiscan FC plate spectrophotometer (Thermo Scientific, Waltham, MA, USA). Cell viability was calculated using optical density (OD) as OD_treated_/OD_control_ × 100%. Three independent experiments were performed for each concentration. Standard deviations did not exceed 10%.

### 2.10. Dynamic Light Scattering Measurements

Particle size distribution histograms in samples at different stages of IONPs treatment and storage were measured three times using the Zetasizer Nano-ZS instrument (Malvern, UK) at a detection angle of 173° and temperature of 25 °C. Each measurement was divided into 10 runs. The runs that contained the poorest data were automatically rejected by the software, while the remaining runs were analyzed and used in the final measurement calculation.

### 2.11. Electron Magnetic Resonance Measurements

IONPs demonstrating in general case ferromagnetic and superparamagnetic nature can be characterized by electron magnetic resonance (EMR) spectra combining signals of ferromagnetic resonance (FMR) and electron spin resonance (ESR) (also known as electron paramagnetic resonance (EPR)) Therefore, concentrations of FAMs and control samples with zero mFA content (HSA@IONPs) and zero HSA content (IONPs) were measured using EMR techniques. EMR spectra were recorded at 25 °C using the X-band spectrometer Bruker EMX-8/2.7 (Ettlingen, Germany). The samples with concentration of IONPs not exceeding 0.05 mg/mL were placed into a resonator of the spectrometer using glass capillaries 1.0 ± 0.1 mm i.d., and the first derivative of the absorption signal was obtained. The measurements were carried out with the following instrumental settings: the microwave power of 6.5 mW, the modulation amplitude of 0.5 mT, the magnetic field resolution of 2048 points, the time constant of 40.96 ms, and sweep time of 167.77 s. EMR spectra of the samples were recorded in magnetic fields of 50–550 mT.

The position of the spectrum center (the resonance field (H_c_) and g-factor) was determined for the samples at different stages of IONPs treatment. The initial hydrosol, after removal of the largest particles with known concentration, was used in a diluted form as the reference data for evaluation of the IONP concentration in the samples.

Mathematical processing of the EMR spectra was carried out using the Bruker (WINEPR V2.22Rev.14) software.

### 2.12. UV/Vis Spectroscopy and Colorimetric Tests

All the samples were analyzed using a UV/Vis spectrometer SPECTROstar Nano (BMG Labtech, Ortenberg, Germany) in 96- and 384-well plates from Greiner, 96-well plates from Costar.

Hydroxyl radical generation by the samples was estimated in colorimetric test with the absorbance measurement at the wavelength (λ_max_ = 418 nm) of 2,3-diaminophenazine (DAP) produced due to the oxidation of 0.075 mM o-phenylendiamine (OPD) (P9029, Sigma-Aldrich) by 9.8 mM hydrogen peroxide (95321, Sigma-Aldrich) in the presence of the samples with peroxidase-like activity with concentration of IONPs equal to 4 µg/mL [17,74] at 37 °C. The DAP formation rate (V_max_, M/s) was calculated using the initial linear section of the optical density (OD) change and the extinction coefficient of 13,000 M^–1^cm^–1^ [74]. The relative DAP formation rate V_max_(sample)/V_max_(unmodified IONPs) (%) was then evaluated for samples on the first and eighth days after preparation and compared to the initial (unmodified) IONPs.

Bradford protein assay was used to control the amount of protein on the surface of the hybrid particles and in the supernatants obtained after magnetic separation. HSA solutions with concentrations from 2 to 100 µg/mL and IONPs hydrosol were used to obtain a calibration curve (the dependence of the optical density at the wavelength of 595 nm on HSA concentration in the presence of IONPs).

### 2.13. Fluorescence Measurements

Fluorescence spectra were recorded using a luminescence spectrometer, Perkin Elmer LS 50, with 0.5 nm resolution and a slit width of 10 nm. Excitation wavelength corresponded to 295 nm for the reaction mixture, containing HSA and mFA, and for the control samples of mFA and HSA. All the samples diluted for measurements were incubated for ~60 min after dilution before measurements. An excitation wavelength of 633 nm was used to detect the fluorescent dye spectra of the samples containing Cy5 and the control sample of HSA conjugated to Cy5. All the spectral measurements were carried out at room temperature in 10 mm quartz cells for the experimental series with mFA and 4 mm for the series with Cy5.

### 2.14. General Remarks on the Sample Preparation and Analysis

All the reagents were of analytical grade or higher. All the solutions were prepared in double-distilled water and incubated at 25 °C. To evaluate the accuracy of the experimental methods all the measurements were carried out for at least three parallel independent samples. The obtained data are presented as the means with standard deviation.

## 3. Results

### 3.1. Interaction Between mFA and HSA in Solution

**Figure 3:**
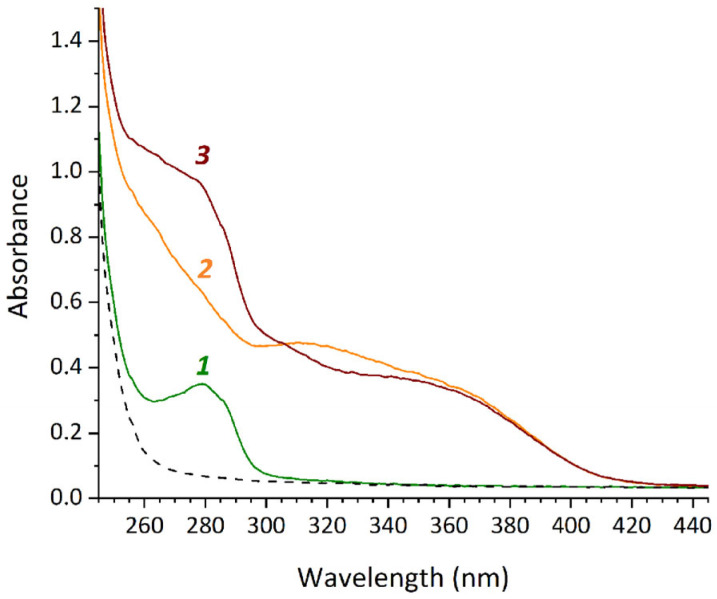
UV/Vis spectra of the samples HSA (1), mFA (2), mFA+HSA (3) with HSA concentration of 13.2 mg/mL, mFA concentration of 0.75 mg/mL (the ratio mFA/HSA equals to 7/1 [mol/mol]) in the mixed solvent (containing 25% DMSO and 25 mM phosphate buffer pH 7.4) diluted after 4 h incubation.

The binding of mFA to HSA in a mixed solvent containing 25% DMSO and water or 25% DMSO and 25 mM phosphate buffer (pH 6.3 or 7.4) was studied using UV/Vis spectrophotometry. Absorption spectra of the sample mFA + HSA and the control samples HSA and mFA were registered. The difference between the spectrum of mFA + HSA and the sum of the spectra of HSA and mFA confirmed binding between the components. The most pronounced changes in the spectra were observed in the wavelength range of 300–325 nm for 25 mM phosphate buffer (pH 7.4) (Figure 3), while they were detected to the less extend for water and 25 mM phosphate buffer pH 6.3.

In the mixed solvent containing 25% DMSO and 25 mM phosphate buffer pH 7.4, the samples of HSA, mFA and mFA + HSA were incubated for different times (10 min, 30 min, 2 h, 4 h, 24 h). It was shown that the representative changes in UV/Vis spectra occur during incubation for more than 2 h. With an increase in the duration of incubation to 24 h, the change in the superposition of the spectra of the components is more pronounced.

**Figure 4:**
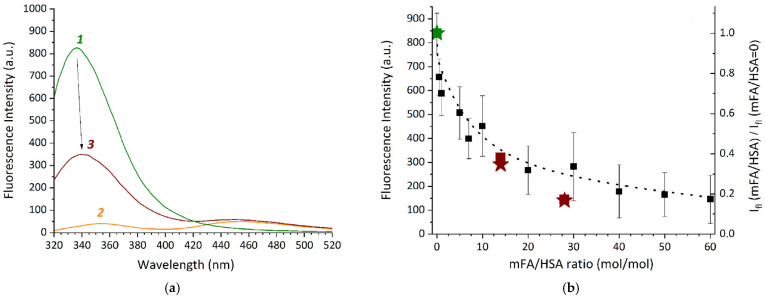
(**a**)—Fluorescence spectra (λ_ex_ = 295 nm) for the samples prepared in the mixed solvent (containing 25% DMSO and 25 mM phosphate buffer pH 7.4) and diluted after 2 h incubation: HSA (1), mFA (2), mFA+HSA (3) (HSA concentration of 13.2 mg/mL, mFA concentration of 0.75 mg/mL, the mFA/HSA ratio equals to 7/1 [mol/mol]); (**b**)—Fluorescence intensity dependence on mFA/HSA ratio at 344 nm for mFA + HSA (squares) and mFA + (HSA-Cy5) (asterisks); right axis is the relation of fluorescence intensities for the sample and for the control sample without mFA.

Fluorescence spectra of the reaction systems containing HSA and mFA were also registered after dilution. The influence of the reaction system composition on the spectra of HSA and mFA was analyzed. In the spectra of mFA + HSA and the control samples fluorescence peaks at ~340 and ~450 nm were observed, which were not related to the solvent (Figure 4a). It is known that the emission peaks around ~350 and ~450 nm are typical for mFA [68], while HSA peak attributed to the Trp residue is usually detected at ~340 nm [75,76]. Spectra (Figure 4a) demonstrate a pronounced fluorescence quenching for HSA in the presence of mFA, which confirms direct binding.

The effect of various mFA/HSA ratios on the albumin fluorescence intensity was analyzed at 344 nm (Figure 4b). Since mFA has limited solubility in water, we used mFA concentrations 0.2 mg/mL in reaction systems mFA + HSA in the mixed solvent containing 25% DMSO and 25 mM phosphate buffer pH 7.4. The concentration of HSA in the reaction systems ranged from 100 to 0.83 mg/mL, with mFA/HSA ranging from 0.5 to 60 [mol/mol]. The samples were incubated in the dark for 2 h, diluted to obtain an equal concentration of HSA (0.83 mg/mL) and then immediately diluted 3-fold in the mixed solvent. The control samples of the relevant concentrations (mFA without HSA, HSA without mFA) were prepared in a similar way.

In Figure 4b a gradual quenching of protein (tryptophan, Trp) fluorescence is observed in the component ratio range from 0.5 to 60 mol of mFA per 1 mol of protein with the most pronounced effect within 0.5–10 mFA/HSA. This indicates binding between the components, resulting in changes in the Trp residue microenvironment. Since we use the 7–28-fold excess of mFA during the sample preparation, we can assume that the covalent binding between the Lys residue of HSA and carboxylic group of mFA (see Scheme 1) occurs with more than one Lys residue available at the HSA@IONP surface. However, we also cannot rule out the non-covalent binding between HSA and folic acid residue with the complex formation, which is possible for HSA and FA and results in the Trp fluorescence quenching [75,77]. The Trp fluorescence quenching is observed both for pure HSA (wine squares compared to olive squares in Figure 4b) and HSA pre-conjugated with Cy5 (wine asterisks compared to olive asterisks in Figure 4b). Both Cy5 and mFA are expected to be covalently bound to the same lysine residues, therefore one can conclude that additional binding of mFA to HSA-Cy5 conjugate may occur due to both complex formation and covalent binding since there are 59 lysine residues in HSA that can be still available after Cy5 conjugation to HSA (according to the data on Cy5 amount in Cy5-HSA presented in the Section 2.3).

### 3.2. FAMs Preparation and Characterization

According to the scheme presented in Figure 1 four steps were carried out for engineering of FAMs.

The analysis of stability and integrity of the coating obtained after the final step of FAMs preparation was carried out by IgG addition followed by EMR spectra registration. It was shown [78,79,80] that IONP spectrum center position in magnetic field depends on distance between magnetic particles. Formation of coating on particles leads to an increase in the distance between them and, accordingly, to a shift in the spectrum center position (g-factor). In our previous studies [17,59] we evaluated the coating thickness using this phenomenon.

As shown in Table 1 by the g-factors of FAMs and the control samples, the coating in FAMs has a thickness similar with the coating on IONPs in the control sample without folate. The stability and integrity characteristics of the coating in FAMs are similar to characteristics of the coating in the sample without folate. These statements are based on the comparison of g-factor of aforementioned samples with g-factor of the control sample without HSA. g-factors for the samples from Step 4 after IgG addition (2.152 and 2.148) are higher than those for the control sample without HSA (2.089). Based on these values we can state that HSA forms coating at IONPs surface and protects IONPs surface from direct contact with IgG.

When comparing g-factors with and without IgG from Table 1 to the data presented in our previous work [17], we can conclude that the differences between the g-factors for the samples of HSA-functionalized IONPs with and without IgG are almost equal (0.023 in the previous study and 0.019 in the current one). Thus, the HSA coatings obtained in the present study and previously can be expected to be similar in their stability and integrity parameters. In the current study, the g-factors for HSA-functionalized IONPs are higher than those detected before. This effect may occur, for example, due to different diameters of the particles and different local magnetic fields in the hybrid particles.

**Figure 5:**
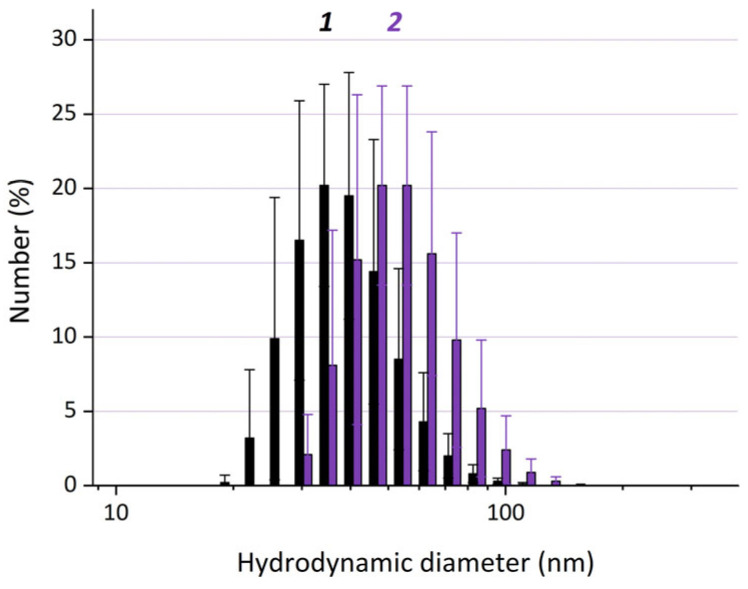
Dynamic light scattering (DLS) size distribution by number for the particles in the control sample IONPs (1) and FAMs (the sample FA-HSA@IONPs) (2). The differences are statistically significant (*p* < 0.05).

The average hydrodynamic diameters of the particles that have the maximal contribution to the number distributions (d_N_) are ~33 ± 2 nm for IONPs without coating and ~54 ± 6 nm for FA-HSA@IONPs obtained at Step 4 (Figure 5). The thickness of the protein coating on IONPs, as calculated from the average hydrodynamic particle diameters (assuming that the coating is formed on IONPs that are ~33 ± 2 nm) is ~11 nm. If the hybrid particles consist of two or more IONPs, the thickness of the protein coating on IONPs is lower and can hardly be quantitatively evaluated.

The interaction of HSA with IONPs at Step 1 was confirmed using UV/Vis absorption and fluorescence spectroscopy using the control sample of HSA and the supernatant obtained at Step 2 after the first stage of magnetic separation. According to the spectra, more than 526 µg of HSA adsorbed on 1 mg of IONPs. The HSA content in the samples of albumin-functionalized IONPs was also evaluated by means of Bradford protein assay after Steps 2 and 4 (before and after interaction with 25% DMSO). After Step 2, the HSA content on IONPs surface was about 193 ± 26 µg protein per 1 mg of IONPs. At Step 4 the HSA content was about 107 ± 32 µg protein per 1 mg of IONPs that could illustrate HSA coating desorption during the multi-step process of FAMs preparation.

Table 2 summarizes main characteristics of FAMs obtained using different physical-chemical methods, and some details of the FAMs characterization are described below. The relative peroxidase-like activity of IONPs in the control sample and in FAMs in the reaction of o-phenylenediamine (OPD) oxidation in the mixture containing IONPs, OPD and hydrogen peroxide with formation of 2,3-diaminophenazine were evaluated by V_max_ at the first day and the eighth day after preparation of the samples. The colorimetric test showed decrease of V_max_ during storage of IONPs for seven days from 100% to 58 ± 16%. The value of the relative speed V_max_(FAMs)/V_max_(IONPs, 1st day) at the first and eighth days were 65 ± 6% and 66 ± 5%, respectively. Thus, we conclude that FAMs could possess peroxidase-like activity when administered to the cells.

To verify the stability and integrity of the HSA coating, the sample of (HSA-Cy5)@IONPs was incubated in 0.05 M phosphate buffer solution pH ~6.3 containing different concentrations of HSA and IgG as described in Section 2.2. According to the data obtained using UV/Vis absorption and fluorescence spectroscopy, the desorption of HSA-Cy5 from (HSA-Cy5)@IONPs does not exceed ~5 µg/mL (~5%) in buffer solution without addition of proteins, while in the presence of extra HSA or IgG it does not exceed ~ 7 µg/mL (~6%) at an initial HSA concentration of ~115 ± 9 µg/mL in this sample. Therefore, the HSA coating could be considered stable and mostly keeping its integrity even after IgG and HSA addition.

### 3.3. Cellular Uptake of (HSA-Cy5)@IONPs and FA-(HSA-Cy5)@IONPs

Magnetic nanoparticles coated with HSA with or without folic acid residue on the surface ((HSA-Cy5)@IONPs and FA-(HSA-Cy5)@IONPs) were accumulated by MCF-7 and MDA-MB-231 breast carcinoma cells. The cell lines selected for the study transcribe folate receptors (FRs) to varying degrees. MDA-MB-231 cells have a high amount of FRs (68%), while MCF-7 cells seem to have very low functional FRs (3%) [81]. The fluorescence of Cy5 covalently attached to albumin on the surface of nanoparticles makes it possible to detect nanosystems inside the cell with fluorescent methods.

**Figure 6:**
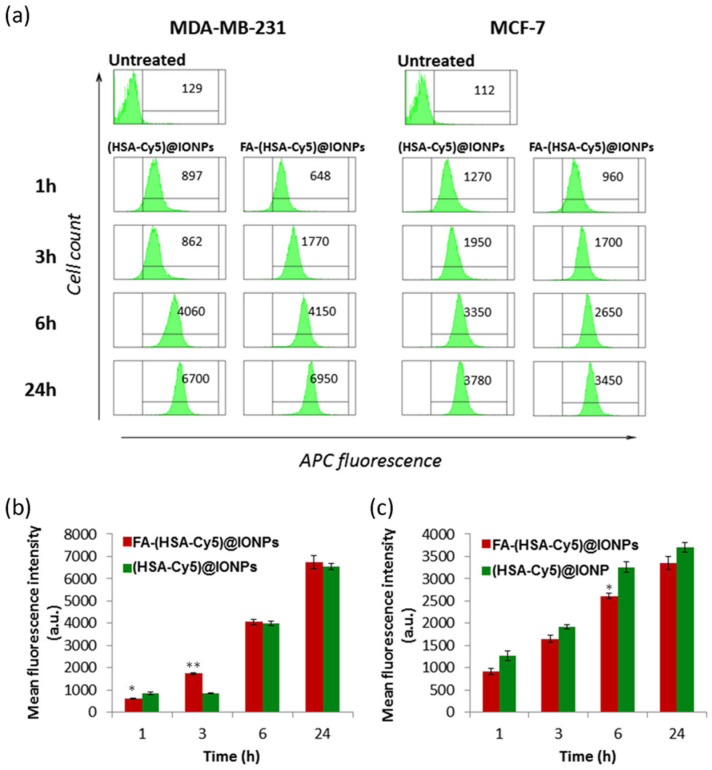
Histograms of (HSA-Cy5)@IONPs and FA-(HSA-Cy5)@IONPs intracellular fluorescence according to the results of flow cytometry via APC channel (**a**). Mean values of MDA-MB-231 and MCF-7 probes ((**b**) and (**c**) respectively). * *p* < 0.05, ** *p* < 0.01 compared to (HSA-Cy5)@IONPs.

We analyzed the cellular uptake kinetics of particles (HSA-Cy5)@IONPs and FA-(HSA-Cy5)@IONPs) at non-toxic concentrations (no toxicity after 24 h, see Section 3.4) by flow cytometry after their incubation with cells for 1–24 h. It was found that right after 1 h of incubation, the hybrid particles both with and without folate begin to penetrate the cells of both cell lines, and by 6–24 h all the cells in the population accumulated the particles (Figure 6).

**Figure 7:**
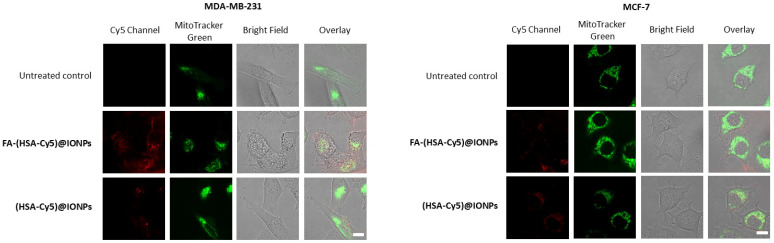
Confocal microscopy of breast carcinoma cells that accumulated the hybrid particles for 3 h. Bar, 10 µm.

The nanosystems with folate (FA-(HSA-Cy5)@IONPs) after 3 h of incubation with MDA-MB-231 cells gave a twofold higher fluorescent signal than nanosystems without folate ((HSA-Cy5)@IONPs). No such difference in accumulation was observed for the MCF-7 cell line. Thus, folic acid residues on the surface of the nanosystems allow them to penetrate more actively into cells with more folate receptors by 3 h of incubation than into cells with a minimal presence of the delivery target. The role of folic acid on the surface of systems as a vector for delivery to cells with a high number of folate receptors was confirmed by analyzing the accumulation of such particles in excess folic acid (Figure S2). To visualize nanosystems inside the cells rather than on the cell surface, we evaluated the fluorescence intensity of nanosystems accumulated by cells of both lines in confocal microscopy (Figure 7).

After 3 h of incubation, the hybrid particles were distributed in the cytoplasm of the cells. Colocalization of the hybrid particles with one of the organelle trackers, the mitochondrial dye MitoTracker Green, was insignificant. After 3 h of incubation, the fluorescence of Cy5 covalently attached to the coating of nanoparticles in FA-(HSA-Cy5)@IONPs sample was significantly higher than for (HSA-Cy5)@IONPs in MDA-MB-231 cells or than for both samples in MCF-7 cells. Thus, to assess the potential of nanosystems with folate group for delivering drug compounds, such as a photosensitizer, into cells, we chose an incubation time of 3 h for these breast carcinoma cell lines.

### 3.4. FAMs as a Photosensitizer Delivering Platform for PDT-Induced Cell Death

To study the potential of the hybrid nanosystems for delivering PSs to cells for in vitro PDT, we chose a well-known photosensitizer, methylene blue (MB) [82], with a high quantum yield of singlet oxygen (Φ_Δ_ = 0.50 in water [83]). The cationic nature of this PS allows MB to be incorporated into protein-coated nanoparticles, especially those functionalized with folate, since electrostatic interactions play an important role in the binding between MB and HSA [84]. The possibility of incorporating methylene blue into this kind of particles also comes from the ability of some PSs to bind non-covalently to albumin [42,43,44]. Methylene blue is known to bind to human serum albumin at sites I (subdomain IIA) and II (subdomain IIIA) [85,86] with an association constant of 4–5 × 10^4^ M^−1^ (ref. [84,85,86]). We carried out the reaction of MB binding with the hybrid particles (FA-HSA@IONPs and HSA@IONPs) according to the method described earlier [87] modified by using magnetic separation. The synthesized particles (MB/FA-HSA@IONPs, MB-HSA@IONPs) contained 5.8 ± 0.6 µg of methylene blue per 1 mg of IONPs (LC% was 0.58 ± 0.06%). Singlet oxygen generation was confirmed using singlet oxygen probe 1,3-diphenylisobenzofuran (Figure S3).

We analyzed the dark and photoinduced cytotoxicity of the hybrid particles with folate on the surface and with non-covalently bound methylene blue after 3 h of accumulation by MCF-7 and MDA-MB-231 cells with further replacement of the cell medium with fresh medium and photoexcitation of the nanosystems with the photosensitizer accumulated by the cells.

**Figure 8:**
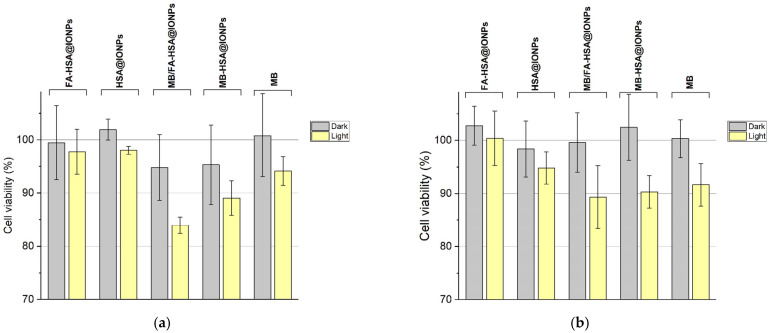
Dark and photoinduced cytotoxicity of the experimental and control samples. (**a**)—viability of MDA-MB-231 cells. (**b**)—viability of MCF-7 cells.

According to the results of the MTT test, all the synthesized nanosystems do not possess dark cytotoxicity for both MDA-MB-231 and MCF-7 cells (Figure 8). When excited by light, MB/FA-HSA@IONPs particles caused greater death of MDA-MB-231 cells than methylene blue in equivalent concentrations serving as a control and MB-HSA@IONPs. This confirms that more efficient delivery of PS into cells is due to the functionalization of the particles with folate, which improves cellular uptake (Figure 6). Additionally, MB/FA-HSA@IONPs exhibit slower MB release from the surface to cell medium under incubation conditions than MB-HSA@IONPs (Figure S4). MB/FA-HSA@IONPs, MB-HSA@IONPs, and MB equally damaged MCF-7 cells, thus confirming that at a low content of folate receptors in cells, folate-modified nanosystems do not have an advantage as a platform for drug delivery over nanosystems without modifications.

Therefore, folate-modified nanosystems can deliver non-covalently albumin-bound PSs with high binding constant (for example, methylene blue) to cells with folate receptors within a few hours (3 h) of incubation. They can be used as a platform for targeted delivery of drugs to cancer cells with a high level of target transcription.

## 4. Discussion

Over the past decades, magnetic iron oxide nanoparticles have been widely used in research aimed at engineering functional materials for biomedical purposes. At the same time, the possible toxic effects of the nanoparticles in the body limit their clinical use [9,88,89,90,91]. In the intracellular environment functionalized particles (hybrid nanosystems) exhibit some unpredicted properties and may lose their expected effectiveness due to the following: (1) Aggregation of particles under physiological conditions; (2) Formation of a protein corona around the particles (particularly, due to insufficient stability and integrity of the coatings), which changes their properties and reduces the circulation time; (3) Toxicity exhibited by the chemically reactive core or/and coating of the particles; (4) Destruction of the coating due to its insufficient stability (low fixation) or/and distortion of the properties of the functional coating.

**Figure 9:**
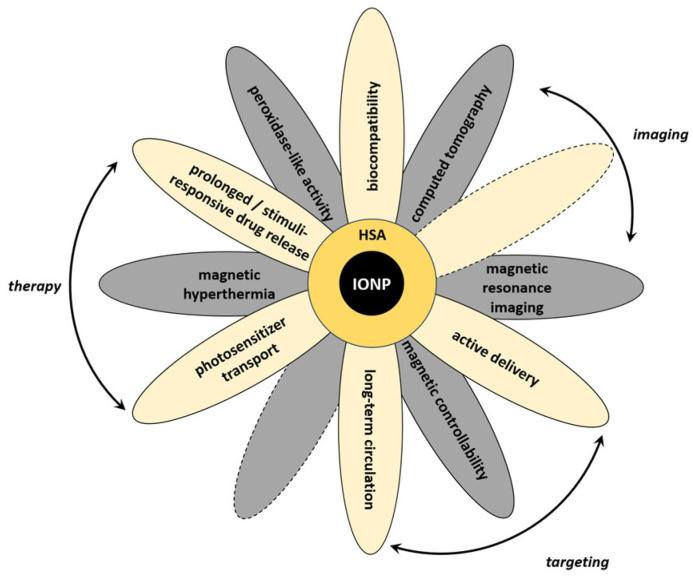
Schematic representation of various properties and applications of hybrid systems based on IONPs and serum albumin.

These effects can be partially reduced for the hybrid nanosystems when serum albumin, the most abundant blood protein, is used as a component of the coating. The albumin coating reduces binding of other serum proteins and IONP toxicity, enhances biocompatibility and blood circulation time for the hybrid nanosystems, and forms new sites for modification with biovectors and drugs. MNPs, particularly IONPs, coated with serum albumin provide many possibilities for using hybrid nanosystems for many medical applications, including theranostics (see Section 1 Introduction and Figure 9). It should be noticed that theranostic application(s) of hybrid nanosystems are often influenced by properties of MNPs. Magnetic controllability of the systems, their contrasting properties, and applicability in hyperthermia may need some tuning of the magnetic properties of nanoparticles for obtaining efficient hybrid systems. Below, we discuss the perspectives of the synthesized nanosystems based on the experimental data presented in this study and information from the literature.

Many papers on the creation of albumin-coated magnetic iron oxide nanoparticles report that the coatings can be fixed on the IONP surface by physical adsorption [92,93]. In our previous works, we developed an original approach to analyzing in vitro whether the albumin coating on the IONP surface is fixed and prevents IONPs from interacting with IgG, taken as a model protein. This approach is based on aggregation of IONPs with unstable albumin coatings, or coatings with decreased coverage of the IONP surface, or without albumin coating in the presence of other blood proteins: fibrinogen [57] or immunoglobulin G [17,59]. Although we have earlier engineered stable protein coatings on the surface of IONPs using free radical processes [17,57], in the current study, we obtained a stable coating from serum albumin without its free radical modification and covalent crosslinking. The amount of protein in FAMs (folate-modified albumin-functionalized magnetic iron oxide nanoparticles) was detected as equal to 107 ± 32 µg per 1 mg of IONPs.

We have analyzed the stability and integrity of the obtained albumin coating when exposed in solution to other protein molecules (HSA, IgG). The albumin coating has also been quantitatively characterized in terms of HSA desorption (the results were obtained using HSA conjugated to Cy5). Using fluorescence spectroscopy and the Bradford method, we confirmed that less than ~6% of HSA could desorb from IONPs into the solution in the presence of the proteins. This amount was close to the corresponding value in the buffer and to the experimental error. The g-factor measurements in EMR experiments have also shown the absence of significant adsorption of IgG. These experiments demonstrated sufficient stability and integrity of the HSA coating. Based on the experiments we suppose HSA coating to have rather high surface coverage and stable fixation on IONPs. Stability and integrity of the coating are of crucial importance for the engineering of hybrid nanosystems for theranostics and their future in vivo applications. It is also noteworthy that the presence of the residual organic solvent may cause additional protein desorption, and hence, the influence of even small amounts of DMSO requires further investigation in the engineering of albumin-functionalized IONPs for medical applications. In our work, DMSO concentration in the hydrosol of FAMs and the corresponding control samples is less than 3% in stocks and less than 0.3% in culture medium.

Peroxidase-like properties are known to often increase the IONP toxicity, but at the same time, to catalyze the formation of reactive oxygen species (ROS) in vivo, causing cellular damage, and thus may be effective in inhibiting tumor growth via ferroptosis [23,94]. To date several strategies are aimed at overcoming hypoxia in the tumor microenvironment which strongly limits the efficiency of PDT due to the lack of the available molecular oxygen [95,96]. Utilizing enzyme-mimetic systems and oxidative stress typical for cancer cells is one of the strategies [97,98]. Peroxidase-mimetic nanozymes are known to be effective in oxygen-dependent cancer phototherapeutics, including PDT [99], while catalase-mimetic nanosystems provide an additional source of molecular oxygen for the ROS production [100,101]. Hence, both peroxidase and catalase activity inherent to IONPs can significantly enhance the photodynamic efficiency of the nanosystems containing photosensitizers. We confirmed the participation of FAMs in hydroxyl radical generation at a physiological temperature of 37 °C compared with the uncoated particles (IONPs) during at least 8 days of storage. It should be noted that peroxidase-like activity of IONPs occurs despite the free radical trapping activity of HSA in blood serum [102,103]. Since we did not use hydrogen peroxide to fix HSA on the surface of IONPs in this study, we could expect the maximal peroxidase-like properties possible for our IONPs. The effect of enhanced penetration and retention (EPR), often referred to as passive targeting, can occur for hybrid nanosystems in tumor tissues. Active particle delivery strategies are mostly based on their preliminary covalent modification with biologically active substances (biovectors), which can bind to the target receptors in the body. Active targeting increases the selective accumulation of the multifunctional hybrid nanosystems. We suppose that for FA-HSA@IONPs, the accumulation under magnetic field exposure is also possible. Therefore, for FAMs (FA-HSA@IONPs) the field-assisted, passive and active delivery strategies can be combined to effectively transport drugs/PSs to cancer cells and provide their controlled release/action. Folic acid (folate) is one of the suitable biovectors specific for many cancer cells. Receptors for folic acid residues are overexpressed by several types of tumors, such as testicular and ovarian cancer, nasopharyngeal tumors, breast cancer, colon cancer, etc. [51,81,104,105]. Currently, the results of preclinical studies suggest that PDT is a promising and effective approach for breast cancer treatment [106]. Breast cancer has a high degree of heterogeneity, and the expression of folic acid (folate) receptors varies in different cell lines. Therefore, we aimed to study the cytotoxic and phototoxic effects of hybrid nanosystems on two different cell lines: MDA-MB-231 with high (68%), and MCF-7 with low (3%) folate receptor expression [81]. The accumulation of FA-HSA@IONPs in the aforementioned cancer cells has been analyzed by means of flow cytometry and confocal laser scanning microscopy. FAMs demonstrated enhanced penetration into the cells with the increased folate receptor expression.

Serum albumin is well known to provide diverse opportunities for further modification of the hybrid particles. A lot of papers analyzing the binding of folic acid to albumin have been published, but very few studies are devoted to the detection of interaction between mFA (NHS-ester of folic acid) and HSA in solution using fluorescence and UV/Vis absorbance spectroscopy [107,108]. In the current study, we used these methods to demonstrate the interaction between mFA and HSA. We expect that binding between mFA and HSA could depend on the mFA/HSA ratio and occur due to both complex formation and covalent linkage. The presence of different binding sites in the albumin molecule is specific for certain biologically active compounds, allowing complexation of different diagnostic and therapeutic agents, including those for photo- and chemotherapy of cancer, while the diversity of the functional groups on the protein surface provides ample opportunities for the covalent modification of the particle coating with different biovectors.

Thus, combination of covalent and non-covalent strategies in the HSA-coated nanoparticle functionalization results in the production of multifunctional theranostic agents. We assume that other biologically active substances (including PSs [109,110]) can also be linked to the FAMs using the multi-step approach developed in the study for mFA. Our future research will be aimed at quantitative characterization of the binding between mFA and HSA@IONPs as well as at increasing the specificity of the FAMs accumulation in the cancer cells, while in the current study, the amount of folate residues in FAMs is less than 20 µg per 1 mg of IONPs.

The current work has been devoted to studying the perspectives of using FA-HSA@IONPs as a delivery platform for photosensitizers in the cancer cells. In this study we tested the effect of PS (methylene blue) non-covalently bound to HSA@IONPs (~5.8 ± 0.6 µg of methylene blue per 1 mg of IONPs). Photoinduced cytotoxicity of the engineered hybrid systems was confirmed, while dark cytotoxicity of MB in the experiments was insignificant. Since MB as a model PS preserves its function, we consider our albumin-functionalized IONPs as a promising platform for PS delivery. The results obtained on the model photosensitizer MB, non-covalently bound to the protein shell of particles, can be significantly improved by a rational choice of PS: with a higher ability to bind to the protein shell of magnetic nanoparticles, with a higher efficiency of ROS generation, as well as with a rational choice of the functionalization strategy (covalent/non-covalent). The model PS demonstrated satisfactory binding constants to the albumin shells of particles (4.38 × 10^4^ for HSA, and 4.04 × 10^4^ for FA-HSA, Figure S1), which can be increased by 1–2 orders of magnitude with a targeted selection of more promising PS than the model one. Non-covalent binding of PS has drawbacks, in particular, uncontrolled drug release from the particle surface, the quantitative assessment of which in these systems depends on the surface modifications, as shown in the additional study in Figure S4. For the rational design of such systems, covalent binding of the PS to the protein shell is also possible, which allows achieving a higher PS loading on particles and more efficient delivery of the PS on particles to cells, detected by standard methods, as shown by us using the example of not PS, but the Cy5 fluorophore (Figure 6, Figure 7 and Figure S2), for which the particle core or surface delivery vectors do not create difficulties in detection. Analysis of ROS as the most important criterion for the effectiveness of PS on a carrier is also possible using generally accepted ROS probes (Figure S3), and this criterion is the most important for choosing the PS itself and obtaining systems that are more photodynamically effective against cancer cells. We also expect HSA binding to reduce the interaction of PS with the magnetic core and prevent PS molecules from aggregation, leading to their stabilization in the photoactive monomolecular form upon complexation with a protein. We suppose that FAMs can also possess peroxidase-like activity when administered to the cells, so we plan to characterize the peroxidase-like activity influence on cancer cells in future studies. However, in the current study, peroxidase-like activity of FAMs appeared to be insignificant (that was confirmed by dark experiments) due to the chosen IONP concentration, experiment duration, insufficient endogenous hydrogen peroxide concentration, etc.

According to our previous work [17], HSA-functionalized IONPs could be present at least for 14 days in inoculated hepatocellular carcinoma PC-1 without immediate or delayed adverse reactions, and no remarkable systemic toxic effects were reported over 14 days of follow-up. Therefore, due to retention of IONPs in blood vessels of tumor [17,111], and the results obtained in the current work, we expect our hybrid nanosystems to be suitable not only for active targeting to folate receptors, but also for the treatment of epidermal diseases [112] and for phototheranostic applications in the treatment of solid cancers [113].

To summarize the data obtained in this study, we have developed hybrid nanosystems FA-HSA@IONPs (folate-modified albumin-functionalized magnetic iron oxide nanoparticles, or FAMs). Using a set of physical-chemical methods (dynamic light scattering (DLS), electron magnetic resonance (EMR) spectroscopy, UV/Vis spectrometry) we proved that we obtained HSA coating on IONPs and quantified the HSA content in the coating and peroxidase-like activity of the particles during 8 days of storage. To ensure folate binding, the experiments were performed in solution using UV/Vis absorption and fluorescence spectroscopy, and the appropriate conditions were chosen for obtaining folate-modified particles. To confirm HSA fixation on the surface of IONPs, we used an original approach based on the affinity of immunoglobulin G to IONPs. To analyze the stability and integrity of the coating and to make the particles visible for flow cytometry and confocal laser scanning microscopy, we used HSA conjugated to Cyanine 5 (Cy5) fluorescent dye. Albumin–Cy5 conjugates were used to show the preservation of the coating in the presence of the protein excess (serum albumin and immunoglobulin G were taken for tests of the coating as the most abundant proteins in the blood plasma). The measurements of intracellular fluorescence of Cy5 covalently bound to albumin on nanoparticles after 1–24 h of incubation showed a folate-dependent accumulation in MCF-7 and MDA-MB-231 breast carcinoma cells, and this contrasting was detected by confocal microscopy. The non-cytotoxic dose of IONPs in the form of HSA conjugates with Cy5 was sufficient for analysis using fluorescent methods, which made it possible to characterize the conditions for the nanosystem uptake into the cell. The nanosystems were shown to have a quantitative advantage in binding to cancer cells with a significant amount of folate receptors during the first three hours compared to HSA@IONPs. Peroxidase-like properties of FAMs were observed during at least 8 days of the hybrid nanosystem storage. The perspectives of using FA-HSA@IONPs in photodynamic therapy were also confirmed. The administration of a photosensitizer capable of non-covalent binding to albumin in the albumin coating made it possible to provide photoinduced cancer cell death while maintaining minimal dark cytotoxicity. Thus, modifying the surface of the particles with folic acid residues enhances their accumulation in cells with overexpression of folate receptors more rapidly than in those with a low content of receptors. A more effective cellular uptake and low dark cytotoxicity of FAMs, together with their increased photoinduced cytotoxicity compared to the non-bound PS (MB) in MDA-MB-231 cells, allowed us to consider our FAMs to be a promising platform for targeted PS delivery.

## 5. Conclusions

The targeted delivery of the protein-bound drugs and photosensitizers (PSs) to the tumors using folate-modified albumin-functionalized magnetic iron oxide nanoparticles (FAMs) is expected to provide targeted therapeutic action and reduce the side effects of these biologically active molecules. We suppose that the combination of passive and active targeting possible for FAMs is a good basis for further performance of the engineered particles in vivo. HSA@IONPs could be useful in different modalities of theranostics (drug and PS delivery, magnetic resonance and fluorescence imaging, photodynamic and photothermal cancer therapy, computed tomography, tumor hyperthermia, etc.) and for multimodal theranostics as well. Essential advantages of the hybrid nanosystems based on IONPs and HSA are: (1) expected applicability for the real-time assessment of the disease progression as well as earlier detection of the disease; (2) magnetically controlled, passive, due to the EPR effect, and active (selective accumulation in the cells with folate receptors) targeting to the cancer cells. Our integrative approach combining covalent and non-covalent nanoparticle modifications opens the prospects to design various multifunctional theranostic agents suitable for different types of cancer.

## Figures and Tables

**Table 1:** Experimental g-factors (±0.006) of the EMR signals of IONPs in the samples with and without immunoglobulin G (IgG).

Sample	Without IgG	With IgG
IONPs from Step 2	2.248	2.089
HSA@IONPs and (HSA-Cy5)@IONPs from Step 2	2.238	2.219
FAMs (FA-HSA@IONPs and FA-(HSA-Cy5)@IONPs from Step 4)	2.200	2.152
HSA@IONPs and (HSA-Cy5)@IONPs from Step 4	2.199	2.148

**Table 2:** The key characteristics of FAMs (FA-HSA@IONPs).

Characteristics	Method	Parameter	Value
Size	DLS	Diameter, nm	~54 ± 6
Peroxidase-like properties	UV/Vis spectrophotometry (Colorimetric detection using o-PDA)	Relative speed V_max_(FAMs)/V_max_(IONPs, 1st day), on the 1st and 8th days after preparation, %	65 ± 6 and 66 ± 5
Concentration	EMR with the reference	IONPs concentration, µg/mL	335 ± 45
Protein amount in the coating	UV/Vis spectrophotometry (Colorimetric detection (Bradford protein assay))	Protein content, µg per 1 mg of IONPs	107 ± 32

## Data Availability

The original contributions presented in this study are included in the article. Further inquiries can be directed to the corresponding author.

## Supplementary Materials

The following supporting information can be downloaded at: https://www.mdpi.com/article/10.3390/pharmaceutics17080982/s1, Figure S1: MB binding studies. (a) Changes in MB absorption spectrum upon addition of HSA (1–11 from 0 to 5 × 10^-5^ M); (b) Benesi-Hildebrand plot for MB and HSA interaction (analytical wavelength 688 nm); (c) Changes in MB absorption spectrum upon addition of FA-HSA (1–11 from 0 to 5 × 10^-5^ M); (d) Benesi-Hildebrand plot for MB and FA-HSA interaction (analytical wavelength 688 nm); Figure S2: Histograms of (HSA-Cy5)@IONPs and FA-(HSA-Cy5)@IONPs intracellular fluorescence according to the results of flow cytometry via APC channel (a). Mean values of MDA-MB-231 probes in the absence and in presence of folate acid excess ((b) and (c) respectively). The differences between “no folic acid” and “folic acid excess” conditions at 3h were statistically significant for (FA-(HSA-Cy5)@IONPs), *p* < 0.01; Figure S3: Singlet oxygen detection with 1,3-diphenylisobenzofuran (DPIBF) in DMSO. (a) DPIBF; (b) HSA@IONPs + DPIBF; (c) MB + DPIBF; (d) MB-HSA@IONPs + DPIBF; (e) Kinetics of DPIBF absorption bleaching at 418 nm. Briefly, a stock solution of DPIBF in DMSO was diluted to a solution with an optical density around 1.0 at 418 nm, after which the studied systems were added. Photoexcitation of MB was performed using a 650 nm laser (5 mW), optical density at excitation wavelength 0.1. All UV-Vis spectra were recorded with a Benchmark Plus Microplate Reader (Bio-Rad Laboratories, Hercules, CA, USA) at room temperature; Figure S4: Methylene blue release kinetics from IONPs in PBS at 37 °C. Briefly, MB-HSA@IONPs and MB/FA-HSA@IONPs were incubated in PBS at 37 °C and magnetically separated at 1, 2 and 3 h, after which MB in the supernatant was analyzed by recording absorbance at 664 nm using a Benchmark Plus Microplate Reader (Bio-Rad Laboratories, Hercules, CA, USA).

## Abbreviations

The following abbreviations are used in this manuscript:

APC	Allophycocyanin
CDT	chemodynamic therapy
Cy5	cyanine 5
DCU	Dicyclohexylurea
DLS	dynamic light scattering
DMEM	Dulbecco’s modified Eagle medium
DMSO	dimethyl sulfoxide
EE%	encapsulation efficiency
EMR	electron magnetic resonance
EPR	electron paramagnetic resonance
EPR	enhanced penetration and retention
ESR	electron spin resonance
FA	folic acid
FAM	folate-modified albumin-coated iron oxide nanoparticle
FMR	ferromagnetic resonance
FR	folate receptor
HSA	human serum albumin
IgG	immunoglobulin G
IONPs	iron oxide nanoparticles
LC%	loading capacity
MB	methylene blue
MCF-7	is the acronym of Michigan Cancer Foundation-7
MDA-MB-231	M D Anderson-Metastatic Breast-231
mFA	NHS-ester of folic acid
MNP	magnetic nanoparticle
MRI	magnetic resonance imaging
MTT	3-(4,5-dimethylthiazol-2-yl)-2,5-diphenyltetrazolium bromide
NHS	N-hydroxy succinimide
OD	optical density
OPD	o-phenylenediamine
PDT	photodynamic therapy
PS	photosensitizer
PTT	photothermal therapy
ROS	reactive oxygen species
UV/Vis	ultraviolet-visible
