# Supplementary material for: Folate-Modified Albumin-Functionalized Iron Oxide Nanoparticles for Theranostics: Engineering and In Vitro PDT Treatment of Breast Cancer Cell Lines
Source: Pharmaceutics. 2025 Jul 30;17(8):982. doi: 10.3390/pharmaceutics17080982 (PMC12389551; doi:10.3390/pharmaceutics17080982)

pharmaceutics

MDPI

Supplementary data

Folate-Modified Albumin-Functionalized Iron Oxide Nanoparticles for Theranostics: Engineering and In Vitro PDT treatment of breast cancer cell lines

Anna V. Bychkova, Maria G. Gorobets, Anna V. Toroptseva, Alina A. Markova, Minh Tuan Nguyen, Yulia L. Volodina, Margarita A. Gradova, Madina I. Abdullina, Oksana A. Mayorova, Valery V. Kasparov, Vadim S. Pokrovsky, Anton V. Kolotaev, Derenik S. Khachatryan

S1. MB Binding studies

The binding interactions between MB and HSA or FA-HSA were studied using UV-Vis spectroscopy. Briefly, to a solution of MB (20 μM in PBS) HSA or FA-HSA was added to various final concentrations (0-50 μM), and the absorption spectra of MB was recorded using a microplate spectrophotometer Benchmark Plus (Bio-Rad Laboratories, CA, USA). The changes in MB absorbance were analyzed using a modified Benesi-Hildebrand equation:

$[A_f-A_0]/[A-A_0] = 1 + 1/(K_b[L])$,

where A_f, A_0 and A are absorbance at saturation, initial absorbance and absorbance at [L]; K_b is the binding constant, [L] is the protein concentration. The binding constant was determined from the slope of the plot of $[A_f-A_0]/[A-A_0]$ against $[L]^{-1}$.

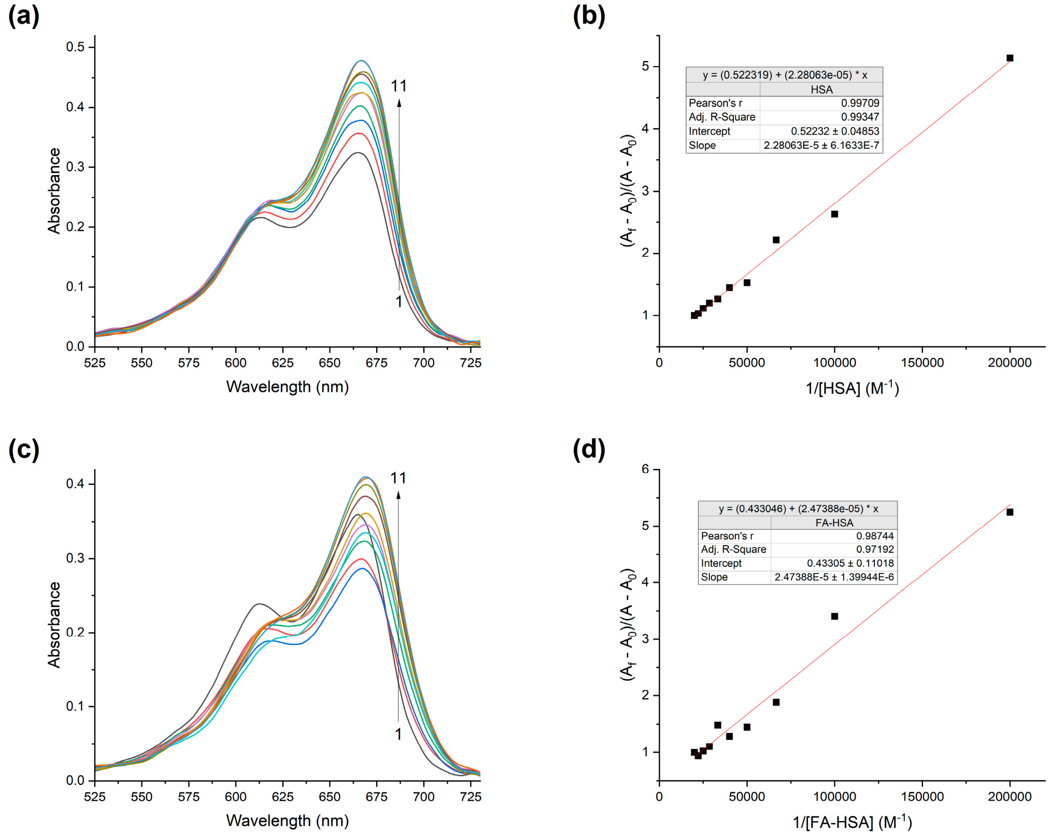

Figure S1. MB binding studies. (a) Changes in MB absorption spectrum upon addition of HSA (1-11 from 0 to 5×10^{-5} M); (b) Benesi-Hildebrand plot for MB and HSA interaction (analytical wavelength 688 nm); (c) Changes in MB absorption spectrum upon addition of FA-HSA (1-11 from 0 to 5×10^{-5} M); (d) Benesi-Hildebrand plot for MB and FA-HSA interaction (analytical wavelength 688 nm).

S2. Cellular uptake of (HSA-Cy5)@IONPs and FA-(HSA-Cy5)@IONPs in excess folate acid

MDA-MB-231 cells grown in 300 µL DMEM in 24-well plates (Nunc, Denmark) were incubated with 30 µL (HSA-Cy5)@IONPs or FA-(HSA-Cy5)@IONPs (FAMs) for 1, 2 and 3 h at 37 °C in excess folate acid obtained from 40 mM DMSO stock solution (final concentration of FA added to the cells was 2 mM) and without FA with addition of DMSO to the cells as a vehicle. After the completion of incubation, the cells were detached with Versene solution at 37°C for 10 minutes and washed with phosphate buffer saline. Fluorescence of the accumulated (HSA-Cy5)@IONPs or FA-(HSA-Cy5)@IONPs was analyzed with flow cytometry on a BD FACS Canto II (BD Biosciences, San Jose, CA) in the APC (Allophycocyanin) channel. For each sample, 10000 events were collected.

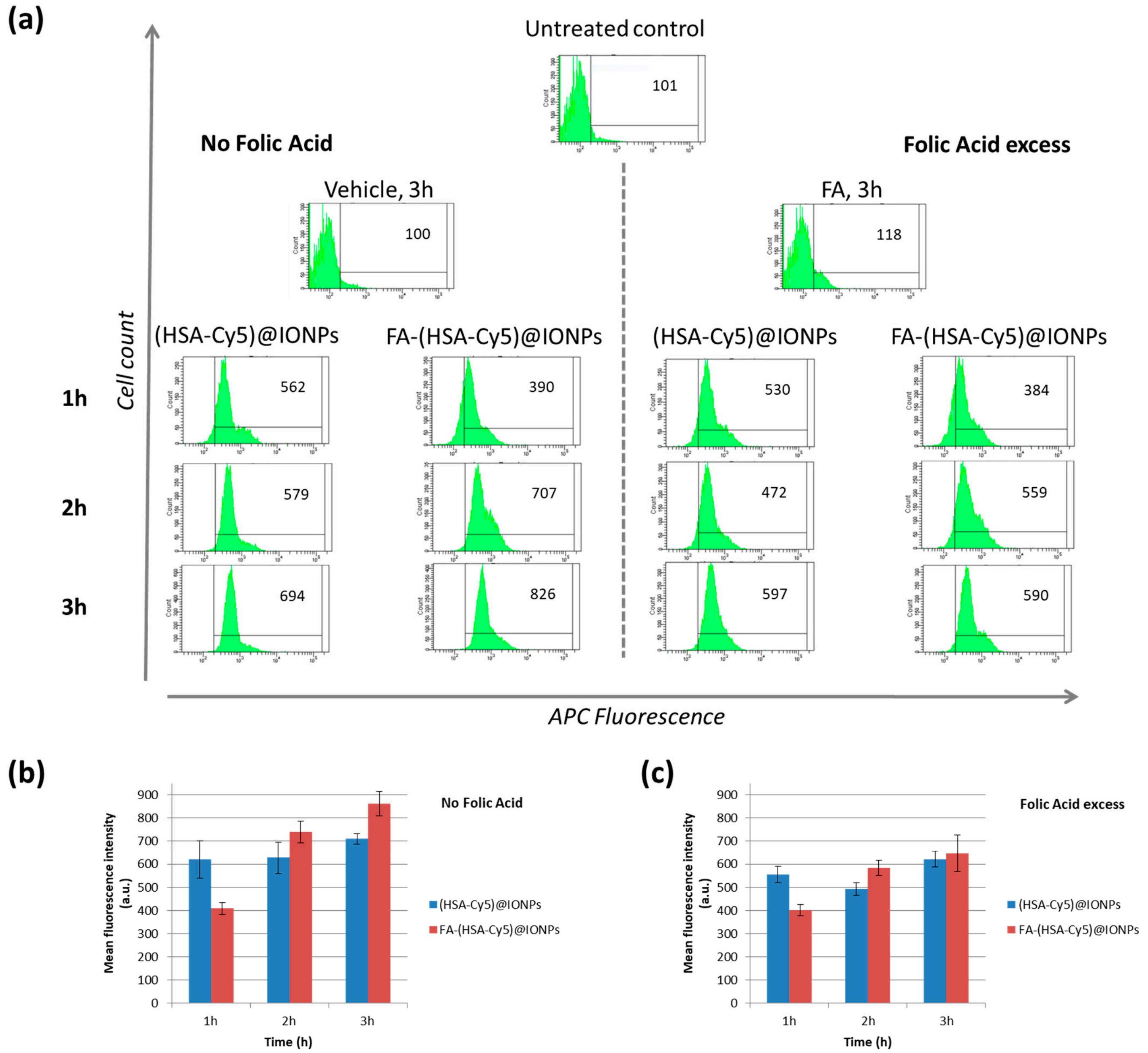

Figure S2. Histograms of (HSA-Cy5)@IONPs and FA-(HSA-Cy5)@IONPs intracellular fluorescence according to the results of flow cytometry via APC channel (a). Mean values of MDA-MB-231 probes in the absence and in presence of folate acid excess ((b) and (c) respectively). The differences between "no folic acid" and "folic acid excess" conditions at 3h were statistically significant for (FA-(HSA-Cy5)@IONPs), $p < 0.01$.

S3. Singlet oxygen detection with 1,3-diphenylisobenzofuran (DPIBF) in DMSO

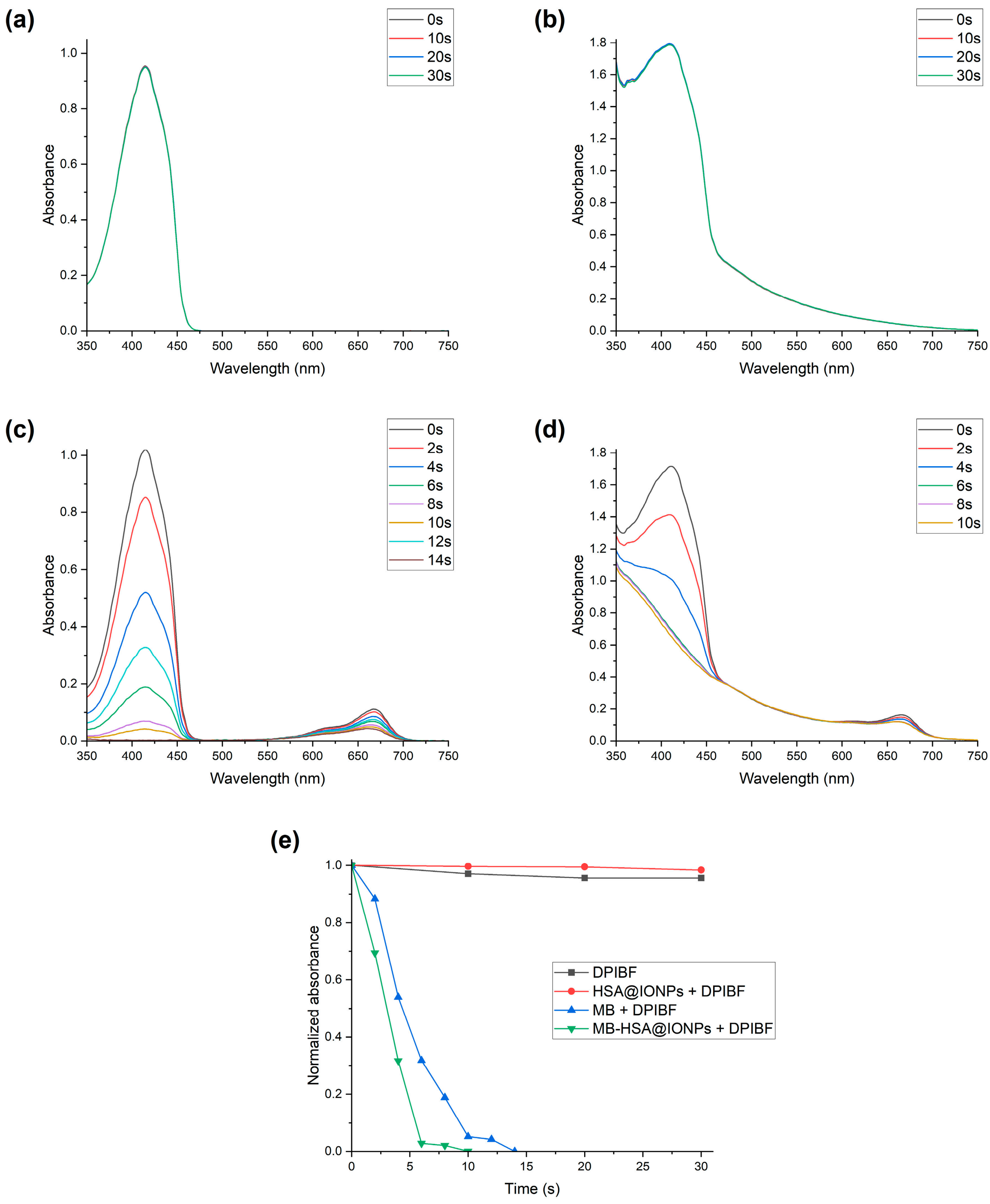

Figure S3. Singlet oxygen detection with 1,3-diphenylisobenzofuran (DPIBF) in DMSO. (a) DPIBF; (b) HSA@IONPs + DPIBF; (c) MB + DPIBF; (d) MB-HSA@IONPs + DPIBF; (e) Kinetics of DPIBF absorption bleaching at 418 nm. Briefly, a stock solution of DPIBF in DMSO was diluted to a solution with an optical density around 1.0 at 418 nm, after which the studied systems were added. Photo-excitation of MB was performed using a 650 nm laser (5 mW), optical density at excitation wavelength 0.1. All UV-Vis spectra were recorded with a Benchmark Plus Microplate Reader (Bio-Rad Laboratories, Hercules, CA, USA) at room temperature.

S4. Methylene blue release kinetics from IONPs with and without FA in PBS at 37°C

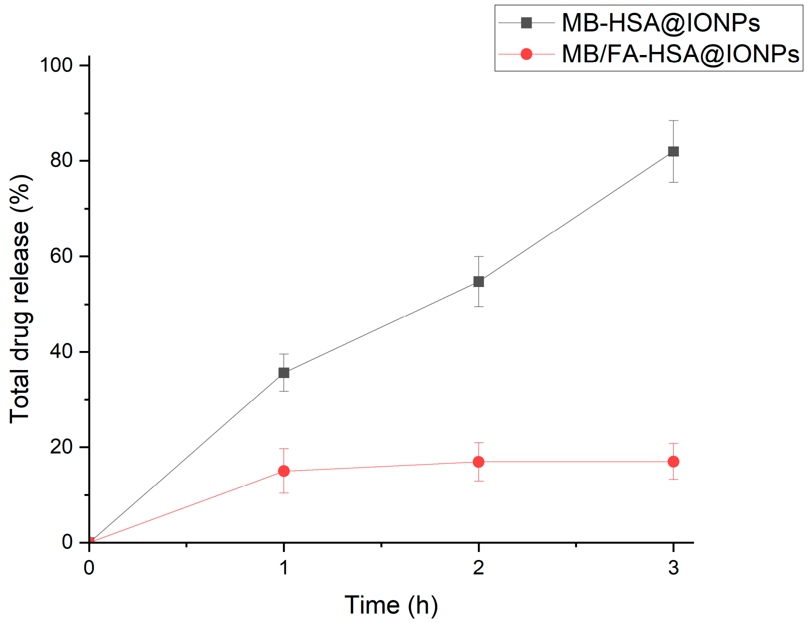

Figure S4. Methylene blue release kinetics from IONPs in PBS at 37°C. Briefly, MB-HSA@IONPs and MB/FA-HSA@IONPs were incubated in PBS at 37°C and magnetically separated at 1, 2 and 3 h, after which MB in the supernatant was analyzed by recording absorbance at 664 nm using a Benchmark Plus Microplate Reader (Bio-Rad Laboratories, Hercules, CA, USA).

S5. DLS parameters for a set of samples: IONPs, HSA@IONPs, FA-HSA@IONPs, MB/FA-HSA@IONPs

The samples from one set of preparation – from IONPs to the complex quaternary structures MB/FA-HSA@IONPs were measured by DLS equipment. The resulting histograms intensity/size and number/size demonstrate low fraction of particles with high sizes. Z-average diameters and PDI values are given below in brackets:

IONPs (Z-average diameter is 82±1 nm; PDI value is 0.186±0.015):

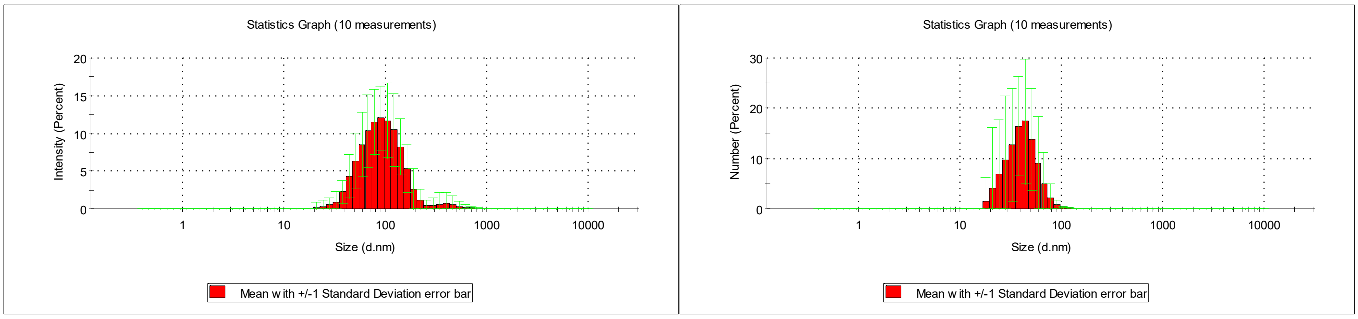

HSA@IONPs (Z-average diameter is 120±1 nm; PDI value is 0.201±0.011):

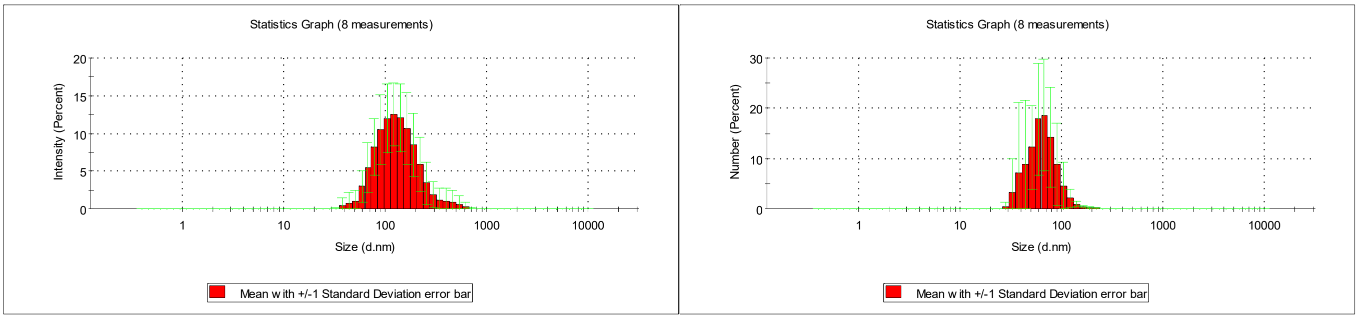

FA-HSA@IONPs (Z-average diameter is 112±2 nm; PDI value is 0.198±0.021):

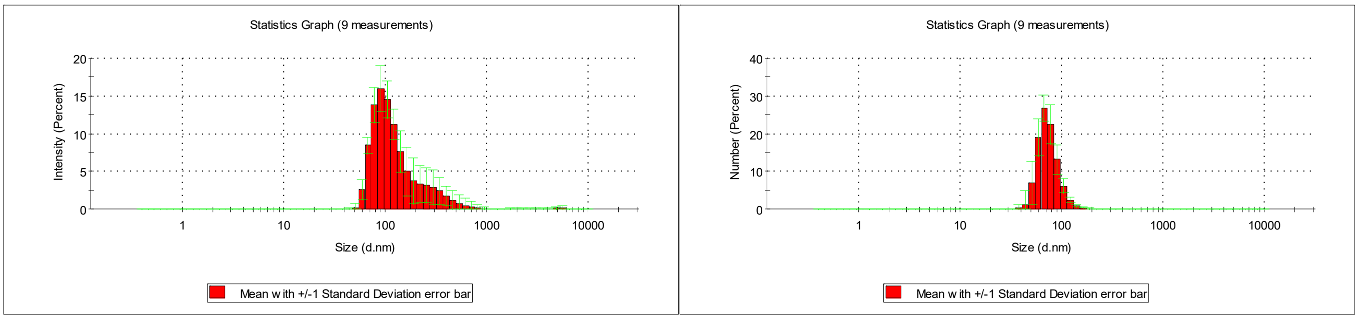

MB/FA-HSA@IONPs (Z-average diameter is 115±2 nm; PDI value is 0.198±0.014):

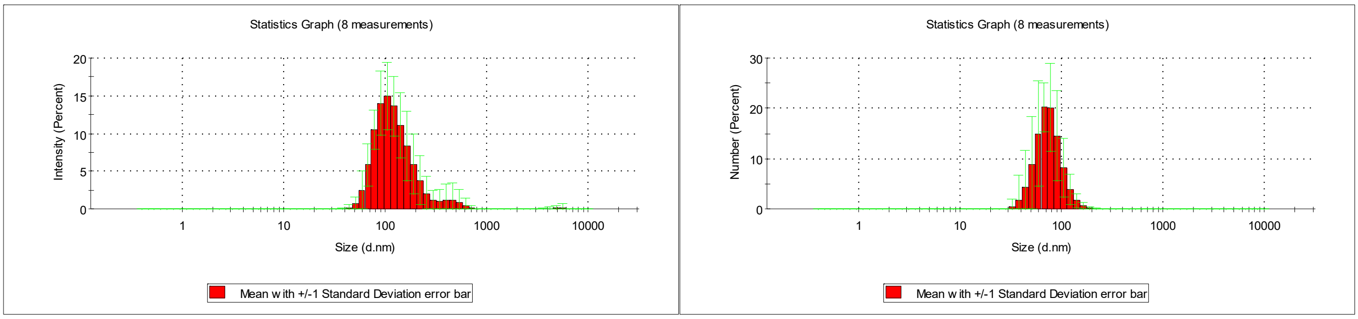

Supplement: Supplementary file 1 [file pharmaceutics-17-00982-s001.zip › pharmaceutics-3717552-supplementary.pdf]
